# Supplementary material for: Cross-linking BioThings APIs through JSON-LD to facilitate knowledge exploration
Source: BMC Bioinformatics. 2018 Feb 1;19:30. doi: 10.1186/s12859-018-2041-5 (PMC5796402; doi:10.1186/s12859-018-2041-5)
Supplement: Supplementary file 1 — A Jupyter Notebook demonstration of how to Make Data-structure Neutral Queries by URI. (HTML 256 kb) [file 12859_2018_2041_MOESM1_ESM.html]

Data-structure Neutral Queries by URI


The actual Jupyter Notebook version could be found here

# 1. Making Data-structure Neutral Queries by URI¶

### Requirements¶

1. Download python package biothings\_client. **biothings\_client** is an easy-to-use Python wrapper to access any Biothings.api-based backend service, including MyGene.info, MyVariant.info, etc. It could be downloaded at pypi or installed using **'pip install biothings\_client'**. In this code demo, we only use functions in **biothings\_client** related to **MyVariant.info**.
2. Downlaod python package requests. **Requests** is the Python HTTP for Humans. It could be installed using **'pip install requests'**.
3. Downlaod python package pyld. **PyLD** is the Python HTTP for Humans. It could be installed using **'pip install PyLD'**.
4. Clone the demo repo and run the code under 'src' folder. **JSON-LD\_BioThings\_API\_DEMO** Repo stores all codes used for the paper. The repo could be found at github. In this demo code, it uses python code **'biothings\_helper'**

##### Use Case Scenario : Query for the OMIM ID for a Variant Using myvariant.info¶

#### Approach 1: Using Myvariant.info¶

Users could retrieve the OMIM ID through myvariant.info; however, it requires the user to understand how OMIM ID is embedded in the data structure of MyVariant.info.

In [3]:

```
# import myvariant python package
from biothings_client import get_client
mv = get_client('variant')
```

In [4]:

```
# Fetch info about OMIM ID
mv.getvariant('chr9:g.135781006_135781007del', fields='clinvar.rcv.conditions.identifiers.omim')
```

Out[4]:

```
{'_id': 'chr9:g.135781006_135781007del',
 '_version': 1,
 'clinvar': {'_license': 'https://goo.gl/OaHML9',
  'rcv': {'conditions': {'identifiers': {'omim': '109800'}}}}}
```

#### Approach 2: Using JSON-LD powered neutral query function¶

By utilizing JSON-LD in making queries, the process would be simplified significantly. Users only need to know the URI for OMIM ID, which is unique for any API. And it saves users significant amount of time in order to figure out the data structure for each API. Code for get\_biothings

In [5]:

```
'''
import get_biothings function which is built utilizing JSON-LD technology
This function could be used to make neutral query for all BioThings APIs,
e.g MyGene.info, MyVariant.info, Drug and compound API
'''
from biothings import get_biothings
```

In [6]:

```
# Fetch info about OMIM ID using URI for OMIM ID, which is 'http://identifiers.org/omim/'
get_biothings(api='myvariant.info',id='chr9:g.135781006_135781007del', fields_uri='http://identifiers.org/omim/')
```

Out[6]:

```
'109800'
```

### Breakdown of get\_biothings function¶

The following code shows each step involved in get\_biothings function demonstrated above.

#### Step1: import JSON-LD context file¶

JSON-LD context file for MyVariant.info

In [7]:

```
import json
import requests
# url for MyVariant.info JSON-LD context
url = "http://myvariant.info/context/context.json"
# load MyVariant.info JSON-LD context file
context = requests.get(url).json()
```

#### Step2: convert uri to field name through JSON-LD context file¶

In [8]:

```
fields_uri='http://identifiers.org/omim/'
field_name = [_field for _field, _uri in context["@context"].items() if _uri==fields_uri]
field_name = ",".join(field_name)
print(field_name)
```

```
clinvar.rcv.conditions.identifiers.omim
```

#### Step3: perform API query¶

In [9]:

```
from biothings_client import get_client
mv = get_client('variant')
mv.getvariant('chr9:g.135781006_135781007del', fields=field_name)
```

Out[9]:

```
{'_id': 'chr9:g.135781006_135781007del',
 '_version': 1,
 'clinvar': {'_license': 'https://goo.gl/OaHML9',
  'rcv': {'conditions': {'identifiers': {'omim': '109800'}}}}}
```
