# Supplementary material for: Cross-linking BioThings APIs through JSON-LD to facilitate knowledge exploration
Source: BMC Bioinformatics. 2018 Feb 1;19:30. doi: 10.1186/s12859-018-2041-5 (PMC5796402; doi:10.1186/s12859-018-2041-5)
Supplement: Supplementary file 2 — A Jupyter Notebook demonstration of how to perform data discrepancy check using JSON-LD. (HTML 263 kb) [file 12859_2018_2041_MOESM2_ESM.html]

Demo for Data Discrepancy Check


The actual Jupyter Notebook could be found here

### This code demonstrate how data discrepancy check is done using JSON-LD¶

### Requirements¶

1. Download python package biothings\_client. **biothings\_client** is an easy-to-use Python wrapper to access any Biothings.api-based backend service, including MyGene.info, MyVariant.info, etc. It could be downloaded at pypi or installed using **'pip install biothings\_client'**. In this code demo, we only use functions in **biothings\_client** related to **MyVariant.info**.
2. Clone the demo repo and run the code under **'src'** folder. **JSON-LD\_BioThings\_API\_DEMO** Repo stores all codes used for the paper. The repo could be found at github. In this demo code, it uses python code **'jsonld\_processor'**.

**jsonld\_processor** is a collection of json-ld related functions. It could be found at the repo. Functions used in this code demo includes **nquads transform** which takes a json-ld doc and transforms it into nquads format. And also **fetch\_value\_by\_uri** which takes an URI, e.g. "http://identifiers.org/dbsnp/" which is the URI for rsid, and return all values in the json-ld doc corresponding to the URI.

## Demo 1: Discrepancy check on rsids.¶

The output of this code is all hgvs ids having rsid discrepancy issues.

In [1]:

```
from biothings_client import get_client
from jsonld_processor import nquads_transform, fetch_value_by_uri, load_context, flatten_doc
import csv
```

In [2]:

```
# count the number of test print ids
test_print = 0
```

In [3]:

```
############################################################################
# Please note looping through all docs in MyVariant.info would take a long
# time. Thus, for demo purpose, we set the limit to the first 200,000 docs.
# You could change the value of total_docs to scan more docs. You could find 
# more hgvs_ids in the output csv file 'rsid_discrepancy_check.csv'.
############################################################################
total_docs = 200000
with open('rsid_discrepancy_check.csv', 'w') as csvfile:
    # count the total number of docs scanned
    cnt = 0
    # json-ld context file for MyVariant.info
    context = load_context('myvariant.info')
    # write the header for csv file
    fieldnames = ['hgvs_id']
    writer = csv.DictWriter(csvfile, fieldnames=fieldnames)
    writer.writeheader()
    # get all docs in MyVariant.info
    mv = get_client('variant')
    data = mv.query(q='__all__', fetch_all=True)
    # loop through each doc, apply jsonld context 
    for doc in data:
        cnt += 1
        if cnt % 50000 ==0:
            print('{} docs have been scanned'.format(cnt))
        # only these sources contain rsid info, so only apply json-ld when one or more these sources appear
        if ('dbnsfp' or 'gwassnps' or 'mutdb' or 'clinvar' or 'dbsnp' or 'evs' or 'grasp') in doc:
            try:
                doc = flatten_doc(doc)
                doc.update(context)
                nquads_doc = nquads_transform(doc)
                rsid = fetch_value_by_uri(nquads_doc, "http://identifiers.org/dbsnp/")
                if rsid and type(rsid) == list:
                    writer.writerow({'hgvs_id': doc['_id']})
                    print_message = doc['_id'] + ': '
                    for i in range(0, len(rsid)):
                        print_message += (str(i+1) + '. ' + rsid[i] + '\t')
                    print(print_message)
            except:
                #print('error id {}'.format(doc['_id']))
                continue
        else:
            continue
        if cnt > total_docs:
            break
```

```
Fetching 424519520 variant(s) . . .
chr15:g.28228629C>A: 1. rs778045887	2. rs147218966	
chr15:g.28953177G>T: 1. rs76825431	2. rs200932053	
chr8:g.12043908A>G: 1. rs2409919	2. rs201884366	
chr8:g.12044020C>T: 1. rs201485916	2. rs200630143	
50000 docs have been scanned
chr11:g.5246838T>A: 1. rs121909829	2. rs33996892	
100000 docs have been scanned
chr1:g.13668975A>G: 1. rs527582969	2. rs61745371	
150000 docs have been scanned
chr10:g.17145142G>C: 1. rs149812870	2. rs2228053	
chrX:g.12712508G>A: 1. rs779596855	2. rs148666498	
200000 docs have been scanned
```

## Demo 2: Discrepancy check on Allele Frequency¶

In [7]:

```
############################################################################
# Please note looping through all docs in MyVariant.info would take a long
# time. Thus, for demo purpose, we set the limit to the first 20,000,000 docs.
# You could change the value of total_docs to scan more docs. You could find 
# more hgvs_ids in the output csv file 'af_afr_discrepancy_check.csv'.
############################################################################
total_docs = 20000000
with open('af_afr_discrepancy_check.csv', 'w') as csvfile:
    # count the total number of docs scanned
    cnt = 0
    # json-ld context file for MyVariant.info
    context = load_context('myvariant.info')
    # write the header for csv file
    fieldnames = ['hgvs_id']
    writer = csv.DictWriter(csvfile, fieldnames=fieldnames)
    writer.writeheader()
    # get all docs in MyVariant.info
    mv = get_client('variant')
    data = mv.query(q='__all__', fetch_all=True)
    # loop through each doc, apply jsonld context 
    for doc in data:
        cnt += 1
        if cnt % 50000 ==0:
            print('{} docs have been scanned'.format(cnt))
        # only these sources contain allele frequency information, so only apply json-ld when one or more these sources appear
        if ('cadd' or 'dbnsfp') in doc:
            try:
                doc = flatten_doc(doc)
                doc.update(context)
                nquads_doc = nquads_transform(doc)
                # please note 'http://identifiers.org/af.afr/' is a placeholder for the URI representing afriacan population allele frequency
                af = fetch_value_by_uri(nquads_doc, "http://identifiers.org/af.afr/")
                if af and type(af) == list:
                    af = [float[_af] for _af in af]
                    af.sort()
                    if af[-1] * 0.5 > af[0]:
                        writer.writerow({'hgvs_id': doc['_id']})
            except:
                #print('error id {}'.format(doc['_id']))
                continue
        else:
            continue
        if cnt > total_docs:
            break
```

```
Fetching 424525377 variant(s) . . .
```
