# Supplementary material for: Cross-linking BioThings APIs through JSON-LD to facilitate knowledge exploration
Source: BMC Bioinformatics. 2018 Feb 1;19:30. doi: 10.1186/s12859-018-2041-5 (PMC5796402; doi:10.1186/s12859-018-2041-5)
Supplement: Supplementary file 6 — A Jupyter Notebook demonstration of BioThings API registry. (HTML 249 kb) [file 12859_2018_2041_MOESM6_ESM.html]

BioThings API registry


The source file including BioThings API registry could be found here

In [1]:

```
from config import AVAILABLE_API_ENDPOINTS
```

In [2]:

```
import pprint
pp = pprint.PrettyPrinter(indent=4)
```

**AVAILABLE\_API\_ENDPOINTS** contains all information about BioThings API registry

In [3]:

```
pp.pprint(AVAILABLE_API_ENDPOINTS)
```

```
[   {   'api': 'mygene.info',
        'input': [   'http://identifiers.org/uniprot/',
                     'http://identifiers.org/ensembl.gene/',
                     'http://identifiers.org/hgnc.symbol/',
                     'http://identifiers.org/wikipathways/',
                     'http://identifiers.org/pubmed/'],
        'jsonld': 'context/mygene_query_context.json',
        'output': ['http://identifiers.org/ncbigene/'],
        'type': 'query',
        'url_syntax': 'http://mygene.info/v3/query?q={{input}}&fetch_all=TRUE&fields=_id'},
    {   'api': 'mygene.info',
        'input': ['http://identifiers.org/ncbigene/'],
        'jsonld': 'context/mygene_context.json',
        'output': [   'http://identifiers.org/uniprot/',
                      'http://identifiers.org/ensembl.gene/',
                      'http://identifiers.org/hgnc.symbol/',
                      'http://identifiers.org/wikipathways/',
                      'http://identifiers.org/pubmed/'],
        'type': 'annotate',
        'url_syntax': 'http://mygene.info/v3/gene/{{input}}'},
    {   'api': 'myvariant.info',
        'input': [   'http://identifiers.org/ncbigene/',
                     'http://identifiers.org/hgnc.symbol/',
                     'http://identifiers.org/ensembl.gene/',
                     'http://identifiers.org/dbsnp/',
                     'http://identifiers.org/pubmed/',
                     'http://identifiers.org/uniprot/'],
        'jsonld': 'context/myvariant_query_context.json',
        'output': ['http://identifiers.org/hgvs/'],
        'type': 'query',
        'url_syntax': 'http://myvariant.info/v1/query?q={{input}}&fetch_all=TRUE&fields=_id'},
    {   'api': 'myvariant.info',
        'input': ['http://identifiers.org/hgvs/'],
        'jsonld': 'context/myvariant_context.json',
        'output': [   'http://identifiers.org/ncbigene/',
                      'http://identifiers.org/hgnc.symbol/',
                      'http://identifiers.org/ensembl.gene/',
                      'http://identifiers.org/dbsnp/',
                      'http://identifiers.org/pubmed/',
                      'http://identifiers.org/uniprot/'],
        'type': 'annotate',
        'url_syntax': 'http://myvariant.info/v1/variant/{{input}}'},
    {   'api': 'mychem.info',
        'input': [   'http://identifiers.org/dbsnp/',
                     'http://identifiers.org/pubchem.compound/',
                     'http://identifiers.org/drugbank/',
                     'http://identifiers.org/pubmed/',
                     'http://identifiers.org/hgnc.symbol/',
                     'http://identifiers.org/uniprot/',
                     'http://identifiers.org/clinicaltrials/'],
        'jsonld': 'context/mychem_query_context.json',
        'output': ['http://identifiers.org/inchikey/'],
        'type': 'query',
        'url_syntax': 'http://mychem.info/v1/query?q={{input}}&fields=_id'},
    {   'api': 'mychem.info',
        'input': [   'http://identifiers.org/inchikey/',
                     'http://identifiers.org/drugbank/'],
        'jsonld': 'context/mychem_query_context.json',
        'output': [   'http://identifiers.org/dbsnp/',
                      'http://identifiers.org/pubchem.compound/',
                      'http://identifiers.org/drugbank/',
                      'http://identifiers.org/pubmed/',
                      'http://identifiers.org/hgnc.symbol/',
                      'http://identifiers.org/uniprot/',
                      'http://identifiers.org/clinicaltrials/'],
        'type': 'annotate',
        'url_syntax': 'http://mychem.info/v1/drug/{{input}}'}]
```

Some of the URIs listed here currently doesn’t exist in ‘identifiers.org’ repo yet. What we put here are name holders for these concepts, e.g. 'http://identifiers.org/ensembl.gene/' and 'http://identifiers.org/clinicaltrials/'. And we are currently contacting ‘identifiers.org’ in terms of adding URIs for these specific terms.
