# Supplementary material for: Cross-linking BioThings APIs through JSON-LD to facilitate knowledge exploration
Source: BMC Bioinformatics. 2018 Feb 1;19:30. doi: 10.1186/s12859-018-2041-5 (PMC5796402; doi:10.1186/s12859-018-2041-5)
Supplement: Supplementary file 7 — A Jupyter Notebook demonstration of how to cross-link BioThings APIs in order to perform complex queries across APIs. (HTML 270 kb) [file 12859_2018_2041_MOESM7_ESM.html]

Crawling API with JSON-LD


The actual Jupyter Notebook version could be found here

## Task Description¶

**Input**: Variant HGVS ID (e.g. chr6:g.26093141G>A)

1. Retrieve Entrez Gene ID(s) **set1g** related to HGVS ID(s)
2. Retrieve Wikipathways ID(s) **set1p** in which Entrez Gene ID(s) set1g are involved
3. Retrieve Other Entrez Gene ID(s) **set2g** which are included in Wikipathways ID(s) set1p
4. Retrieve Uniprot ID(s) **set2u** which correspond to Entrez Gene ID(s) set2g
5. Retrieve Drug Inchi Key(s) **set1d** which target Uniprot ID(s) set2u

**Output**: Available drugs targetting genes/pathways related to the input HGVS ID

### Requirements¶

1. Clone the demo repo and run the code under **'src'** folder. **JSON-LD\_BioThings\_API\_DEMO** Repo stores all codes used for the paper. The repo could be found at github. In this demo code, it uses python code **'biothings\_helper'**, **'biothings'** and **'jsonld\_processor'**.

### Input¶

In [1]:

```
# input a list of variants
variant_list = ['chr6:g.26093141G>A', 'chr12:g.111351981C>T']
```

### Import biothings library¶

In [2]:

```
# IdListHandler class convert a list of IDs from a given input type to another list of IDs from a given output type
from biothings import IdListHandler
ih = IdListHandler()
# dict2list help convert the dictionary output to list
from biothings import dict2list
```

### Step 1: HGVS ID >- Entrez Gene ID¶

In [3]:

```
gene_result = ih.list_handler(input_id_list=variant_list, 
                              _input_type='http://identifiers.org/hgvs/', 
                              _output_type='http://identifiers.org/ncbigene/', 
                              uri=True)
print(gene_result)
set1g = dict2list(gene_result)
```

```
{'chr6:g.26093141G>A': [{'source': 'myvariant.info', 'entrez_gene_id': '3077'}], 'chr12:g.111351981C>T': [{'source': 'myvariant.info', 'entrez_gene_id': '4633'}]}
```

### Step 2: Entrez Gene ID >- Wikipathways ID¶

In [4]:

```
wikipathway_result = ih.list_handler(input_id_list=set1g, 
                                     _input_type='http://identifiers.org/ncbigene/', 
                                     _output_type='http://identifiers.org/wikipathways/', 
                                     uri=True)
print(wikipathway_result)
set1p = dict2list(wikipathway_result)
```

```
{'3077': [{'source': 'mygene.info', 'wikipathway_id': 'WP3924'}], '4633': [{'source': 'mygene.info', 'wikipathway_id': ['WP289', 'WP3888', 'WP383', 'WP2406']}]}
```

### Step 3: Wikipathways ID >- Entrez Gene ID¶

In [5]:

```
gene_result2 = ih.list_handler(input_id_list=set1p, 
                               _input_type='http://identifiers.org/wikipathways/', 
                               _output_type='http://identifiers.org/ncbigene/', 
                               uri=True)
set2g = dict2list(gene_result2)
print(len(set2g))
```

```
453
```

### Step 4: Entrez Gene ID >- Uniprot ID¶

In [6]:

```
uniprot_result = ih.list_handler(input_id_list=set2g, 
                                 _input_type='http://identifiers.org/ncbigene/', 
                                 _output_type='http://identifiers.org/uniprot/', 
                                 uri=True)
set1u = dict2list(uniprot_result)
print(len(set1u))
```

```
457
```

### Step 5: Uniprot ID >- Inchi Key¶

In [7]:

```
inchi_result = ih.list_handler(input_id_list=set1u,
                              _input_type='http://identifiers.org/uniprot/',
                              _output_type='http://identifiers.org/inchikey/',
                              uri=True)
inchi_list = dict2list(inchi_result)
print(len(inchi_list))
print(inchi_list[0:5])
```

```
466
['ACPOUJIDANTYHO-UHFFFAOYSA-N', 'AECPTICWHONWNW-UHFFFAOYSA-N', 'AFSDNFLWKVMVRB-UHFFFAOYSA-N', 'AMNKRBRQQAMACZ-UHFFFAOYSA-N', 'APYXQTXFRIDSGE-UHFFFAOYSA-N']
```

### Breakdown of list\_handler function¶

The following code shows each step involved in list\_handler function demonstrated above using entrez gene id-> wikipathway transformation as an example.

Metadata information about BioThings API(config)

#### Step 1: Specify input and output¶

In [8]:

```
input_value = '3077'
input_type='http://identifiers.org/ncbigene/'
output_type='http://identifiers.org/wikipathways/'
```

#### Step 2: Iterate through API metadata info, and find corresponding API based on input & output¶

In [9]:

```
from config import AVAILABLE_API_ENDPOINTS
from jsonld_processor import flatten_doc
from biothings_helper import construct_url, find_id_from_uri, find_value_from_output_type
```

In [10]:

```
# convert to internal input name and output name
input_name = find_id_from_uri(input_type)
output_name = find_id_from_uri(output_type)
# look up api in api metadata info
endpoint_list = []
for i in range(0, len(AVAILABLE_API_ENDPOINTS)):
    if input_name in AVAILABLE_API_ENDPOINTS[i]["input"] and output_name in AVAILABLE_API_ENDPOINTS[i]["output"]:
        endpoint_list.append(i)
print(AVAILABLE_API_ENDPOINTS[endpoint_list[0]]["api"])
```

```
mygene.info
```

#### Step 3: Make API call¶

In [11]:

```
# construct url based on metadata info
url = construct_url(endpoint_list[0], input_value, input_name)
# make API call
import requests
doc = requests.get(url).json()
#for better display in ipython notebook, we are not printing the whole json doc here
#the following code could be used to display the json_doc returned from api call
# print(doc)
```

#### Step 4: Transform JSON doc to JSON-LD doc and Nquads format¶

In [12]:

```
# load context file
import json
json_doc = flatten_doc(doc)
context = json.load(open(AVAILABLE_API_ENDPOINTS[endpoint_list[0]]["jsonld"]))
# construct json-ld doc
json_doc.update(context)
# transform json-ld doc to nquads format
from pyld import jsonld
t = jsonld.JsonLdProcessor()
nquads = t.parse_nquads(jsonld.to_rdf(json_doc, {'format': 'application/nquads'}))['@default']
print(nquads[1])
# for better display in ipython notebook, we are not printing the whole nquads doc here\
# the following code could be used to display the whole nquads doc
# print(nquads)
```

```
{'predicate': {'value': 'http://identifiers.org/ensembl.protein/', 'type': 'IRI'}, 'subject': {'value': '_:b0', 'type': 'blank node'}, 'object': {'value': 'ENSP00000259699', 'type': 'literal', 'datatype': 'http://www.w3.org/2001/XMLSchema#string'}}
```

#### Step 5: Fetch value using URI from Nquads format¶

In [13]:

```
value_list = []
for item in nquads:
    if item['predicate']['value'] == output_type:
        value_list.append(item['object']['value'])
value = list(set(value_list))
print(value)
```

```
['WP3924']
```
