# Supplementary material for: Cross-linking BioThings APIs through JSON-LD to facilitate knowledge exploration
Source: BMC Bioinformatics. 2018 Feb 1;19:30. doi: 10.1186/s12859-018-2041-5 (PMC5796402; doi:10.1186/s12859-018-2041-5)
Supplement: Supplementary file 8 — A Jupyter Notebook demonstration of how to convert a JSON document from MyVariant.info into RDF using JSON-LD. (HTML 248 kb) [file 12859_2018_2041_MOESM8_ESM.html]

Demo for Serializing RDF from JSON


The actual Jupyter Notebook could be found here

### Retrieve JSON-LD document from MyVariant.info¶

### Requirements¶

1. Downlaod python package Requests. **Requests** is the Python HTTP for Humans. It could be installed using **'pip install requests'**.
2. Download python package PyLD. **PyLD** is the Python implementation of the JSON-LD API. It could be installed using **'pip install PyLD'**

In [1]:

```
import requests
url = 'http://myvariant.info/v1/variant/chr6:g.26093141G>A?jsonld=TRUE'
json_doc = requests.get(url).json()
```

### Import Python library PyLD¶

In [2]:

```
from pyld import jsonld
```

### Serialize MyVariant.info JSON-LD Doc to RDF format¶

In [3]:

```
rdf = jsonld.normalize(json_doc, {'algorithm': 'URDNA2015', 'format': 'application/nquads'})
# print only the first 1040 charcters of the rdf file for simplicity
print(rdf[1:1040])
```

```
http://myvariant.info/v1/variant/chr6:g.26093141G>A> <http://identifers.org/clinvar/> "RCV000000019" .
<http://myvariant.info/v1/variant/chr6:g.26093141G>A> <http://identifers.org/clinvar/> "RCV000000020" .
<http://myvariant.info/v1/variant/chr6:g.26093141G>A> <http://identifers.org/clinvar/> "RCV000000021" .
<http://myvariant.info/v1/variant/chr6:g.26093141G>A> <http://identifers.org/clinvar/> "RCV000000022" .
<http://myvariant.info/v1/variant/chr6:g.26093141G>A> <http://identifers.org/clinvar/> "RCV000000023" .
<http://myvariant.info/v1/variant/chr6:g.26093141G>A> <http://identifers.org/clinvar/> "RCV000000024" .
<http://myvariant.info/v1/variant/chr6:g.26093141G>A> <http://identifers.org/clinvar/> "RCV000000025" .
<http://myvariant.info/v1/variant/chr6:g.26093141G>A> <http://identifers.org/clinvar/> "RCV000117222" .
<http://myvariant.info/v1/variant/chr6:g.26093141G>A> <http://identifers.org/clinvar/> "RCV000178096" .
<http://myvariant.info/v1/variant/chr6:g.26093141G>A> <http://identifers.org/clinvar/> "RCV000210820" .
```
